# The Diseases of Children; a Treatise Intended for the Use of the Student and Junior Practitioner

**Published:** 1842-01

**Authors:** 


Art. V.—*The Diseases of Children; a Treatise intended for the use of the Student and Junior Practitioner.* By Augustus Rees, m.r.c.s., Surgeon to the General Dispensary for Children.—*London*, 1841. 8vo, pp. 300.

Mr. Rees's little volume is, we think, well adapted for the purpose for which it is intended, viz. to serve as a practical guide to the junior practitioner. It is evidently the work of a man of observation and experience, who knows what has been done by others and has tested their experience by his own.

